# Mono–Material 4D Printing of Digital Shape–Memory Components

**DOI:** 10.3390/polym13213767

**Published:** 2021-10-30

**Authors:** Dalia Niazy, Ahmed Elsabbagh, Mostafa R. Ismail

**Affiliations:** 1 Architecture Department, Faculty of Engineering, Ain Shams University, Cairo 11566, Egypt; mostafa_ismail@eng.asu.edu.eg; 2 Design and Production Engineering Department, Faculty of Engineering, Ain Shams University, Cairo 11566, Egypt; elsabbagh.ahmed@eng.asu.edu.eg

**Keywords:** 4D printing, material programming, digital fabrication, shape-memory polymers

## Abstract

Dynamic shading systems in buildings help reduce solar gain. Actuated systems, which depend on renewable energy with reduced mechanical parts, further reduce building energy consumption compared to traditional interactive systems. This paper investigates stimuli-responsive polymer application in architectural products for sustainable energy consumption, complying with sustainable development goals (SDGs). The proposed research method posits that, by varying the infill percentage in a pre-determined manner inside a 3D-printed mono-material component, directionally controlled shape change can be detected due to thermal stimuli application. Thus, motion behavior can be engineered into a material. In this study, PLA+, PETG, TPU and PA 6 printed components are investigated under a thermal cycle test to identify a thermally responsive shape-memory polymer candidate that actuates within the built environment temperature range. A differential scanning calorimetry (DSC) test is carried out on TPU 95A and PA 6 to interpret the material shape response in terms of transitional temperatures. All materials tested show an anisotropic shape-change reaction in a pre-programmed manner, complying with the behavior engineered into the matter. Four-dimensional (4D)-printed PA6 shows shape-shifting behavior and total recovery to initial position within the built environment temperature range.

## 1. Introduction

Digital materiality is defined as the combination of digital and material disciplines in the fields of built environment design and construction [1]. The application of 3D printing digital fabrication to acquire smart spatial structures has been widely investigated [2,3,4,5]. Three-dimensional (3D) printing as an additive manufacturing (AM) method is highly preferred due to the recyclability it allows, which enhances system sustainability [6]. The application of the 3D printing method to acquire stimuli-responsive spatial deformation in a programmed manner has been classified as a 4D printing manufacturing paradigm [7,8]. Four–dimensional (4D)–printed components hold a promising method for advancing interactive architecture components by exploiting surrounding environment parameters—renewable energy resources—as stimuli for motion.

Implementing 4D polymeric material programming in architecture can enable building interactive system components, which can be achieved in six steps: system motion design, identifying the required angle of bending that corresponds with the solar radiation penetration, system component identification, allocation of active components within the system and programming the polymers by 3D printing. Four-dimensional (4D) printing manufacturing parameters are identified as final desired shape, material properties, stimulus and material structure through printing paths [8]. Material structure by bi-/multi-layered 3D-printed multi–material composites is classified as an established method for attaining a 3D stimuli-responsive structural form from 2D structures [9,10,11,12]. The act of recovering a polymer to its initial position after actuation upon stimuli application is identified as a two-way shape-memory polymer (SMP) [4,13,14].

Most two-way shape-memory effects previously achieved in polymers required composite formation [12,15]. Thus, the material structure of multi-materials has been widely investigated. Consequently, the material structure programming parameter through control of printing paths has undergone an evolution, which started from 3D patterning of an actuating polymer above an elastomeric matrix to the complete weaving of multi-materials [12,16,17,18,19,20]. As an example, Zolfagharian et al. [16] achieved different angles and rates of bending of a film component by varying the pattern of chitosan hydrogel ink coated in the middle of a 3D-printed pre-strained polystyrene film. Wang et al. [17] achieved variable deployable structures of bi-layer, multi-material printed components by varying the pattern of carbon fibers on a flexible polyamide 66 matrix. In [21,22], the effect of adding fillers to an SMP enhanced the thermal and/or electric conductivity of the SMP matrix, resulting in reduced actuation and recovery time, as well as reduced electric current consumption in the case of an electro-activated SMP. Correa et al. [23] applied multi-material 4D printing to achieve an architectural-scale product by weaving a composite of wood polymer composite (WPC) with acrylonitrile butadiene styrene (ABS) in different patterns. The weaved composite showed variable directional control in correspondence with 3D-printed patterns angles. It was observed that submerging the SMP matrix, before stimuli application, in solvents modified the glass transition temperature T_g_, which was identified as a plasticizing effect [24,25].

Gladman et al. [26] explored different spatial deformation from 2D to 3D structures through 3D printing nozzle path variation, expressed as patterning for a single material. The single material Galdman et al. used was a digital hydrogel composite. The cellulose fibril alignment inside the hydrogel controlled the motion direction. In general, directional motion control in self-shape-shifting components depends on stress. The shape-memory effect (SME) is defined as the proportional change to stimuli applied upon material programming [14,27]. Thermal stimuli-responsive composite manufacturing is based on volumetric thermal expansion (Equation (1)). Variable volume change due to variance in the thermal expansion coefficient (α) of each lamina/part/layer causes stress between layers, evoking directional motion control, which produces overall composite shape variation in response to stimuli application.

(1) $$\Delta V=3V_{0}\times \alpha \times \Delta T$$

The complexity of 3D printing by fused deposition modeling (FDM) is increased in multi-polymeric materials. A multi-layered component for 4D printing, where the materials’ required print bed temperature may vary, may pose a constraint for printing feasibility. Thus, further investigation of Galdman et al.’s method with a different approach for the printing of single material is conducted in this paper. This paper investigates thermal stimuli-responsive shape shifting by 3D printing of a single/mono-material to acquire anisotropic shape shifting through variable infill parameters during printing. Motion direction control is programmed into the material by applying variable infill parameters of nozzle path design and patterning effect. This verifies the hypotheses that, for a 4D-printed mono-material at a constant thermal expansion coefficient (α), varying the initial volume (V_0_) of the separate layers/lamina of a single/mono 3D-printed material will result in an overall anisotropic volumetric change in a controlled direction and shape. The volumetric change will be induced due to the generation of internal stresses inside the sample directing the motion. Thus, 4D printing of a single/mono-material of variable infill parameters is investigated through experiments. This method presents a different patterning approach. The approach proposed differs from that of Galman et al. in that the directional motion control is achieved using variable infill parameters. As per the experiments conducted, 4D-printed samples can actuate in a preprogrammed manner, as the angle of curvature—motion direction—is affected by the infill pattern, initial volume—infill percentage—of each layer, glass transition range and Young’s modulus of the material.

**Figure 1:**
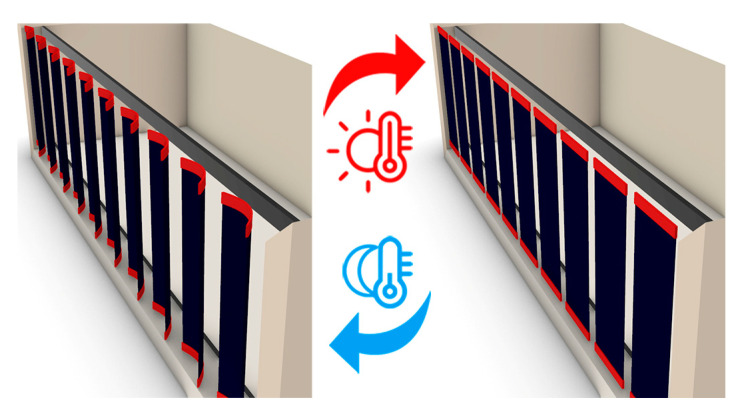
Sun-shading architecture interactive system. It actuates according to heat stimuli.

The computationally simulated dynamic system in Figure 1 is a bending-active system with variable angles of curvature, demonstrating possible architecture application. The system consists of two parts of 4D-printed components as sensors and motion drivers, while a textile sheet is fixed in between. Upon the programming of motion into the material, the material becomes informed to acquire an environmentally passive dynamic system, and the shading unit produced is thermally actuated. Kangaroo in the Grasshopper plugin was utilized for the model motion simulation. The angles of curvature observed from the experiments are used as motion limits in the simulation.

## 2. Materials and Methods

Material selection depended on the commercial availability of environmentally friendly filaments in Egypt. Polylactic acetate (PLA+)(eSUN, Shenzhen, China), polyamide 6 (eSUN PA 6), thermoplastic polyurethane 95A (eSUN TPU 95A) and recyclable polyethylene terephthalate glycol (eSUN PETG) filaments were investigated. Ester–based TPU [28] and PLA [11,28,29] are biodegradable. Thus, eSUN PLA+ filament was used. PLA+ was selected, as it has ten times higher mechanical strength than PLA. PA 6 was selected, as it is of high mechanical strength and biocompatible [29]. Due to the commercial availability of filaments, PA 6 was selected over biodegradable polyamide 11 (PA 11), and eSUN TPU 95A filament was selected over ester–based TPU. The selected filaments’ elongation percentages as received by the supplier are 196%, 12%, 780% and 225% for PA 6, PLA+, TPU 95A and PETG, respectively. Young’s modulus was calculated to be 225.2, 9.054, 12.09 and 6.37 N/mm^2^ for PLA+, PETG, PA 6 and TPU 95A, respectively.

### 2.1. 3D Printing

All materials investigated were 3D printed using FDM 3D printers. PLA+, TPU 95A and PETG were 3D printed using the Creality CR-10 V2 FDM(Creality, Shenzhen, China) printer, while PA 6 was printed using the ANYCUBIC Chiron printer (ANYCUBIC, Shenzhen, China). The PLA+ filament printing parameters are 200 °C, 50 °C and 60 mm/s for nozzle temperature, bed temperature and printing speed, respectively. The PA 6 filament printing parameters are 255 °C, 90 °C and 50 mm/s for nozzle temperature, bed temperature and printing speed, respectively. The TPU 95A printing parameters are 220 °C, 60 °C and 30 mm/s for nozzle temperature, bed temperature and printing speed, respectively. The PETG filament printing parameters are 245 °C, 70 °C and 40 mm/s for nozzle temperature, bed temperature and printing speed, respectively.

In this paper, one of Bakradze et al.’s [30] optimization strategies were used. The low-surface–roughness optimization strategy was utilized to provide higher geometrical precision and good mechanical properties. Printing speed was lowered with higher-temperature printing. Geometrical precision is essential for the investigation of the infill pattern and percentage method, as the material distribution is a key parameter to investigate 4D printed mono-material programmed motion.

### 2.2. Motion Programming Methodology

The directional motion investigation utilized material allocation during 3D printing. The infill variation parameter was suitable for mono-material 4D printing. Programming was performed by designing the print path parameters—infill percentage and pattern—followed by a thermal stimuli application step for the one-way shape-memory effect. In the two-way shape-memory effect, material programming was performed through print path design, while stimuli application was for validation of recoverability. Sample component geometry was computationally modeled using Rhinoceros software. Then, Ultimaker Cura (Software, Utrecht, The Netherlands) was used to control the infill printing parameters, which was the shape–shifting stimulator. Four-dimensional (4D)-printed components were not submerged in any solvent. The 4D mono-material printing method was conducted on different materials with different mechanical parameters to investigate the resulting one-way or two-way SME.

**Figure 2:**
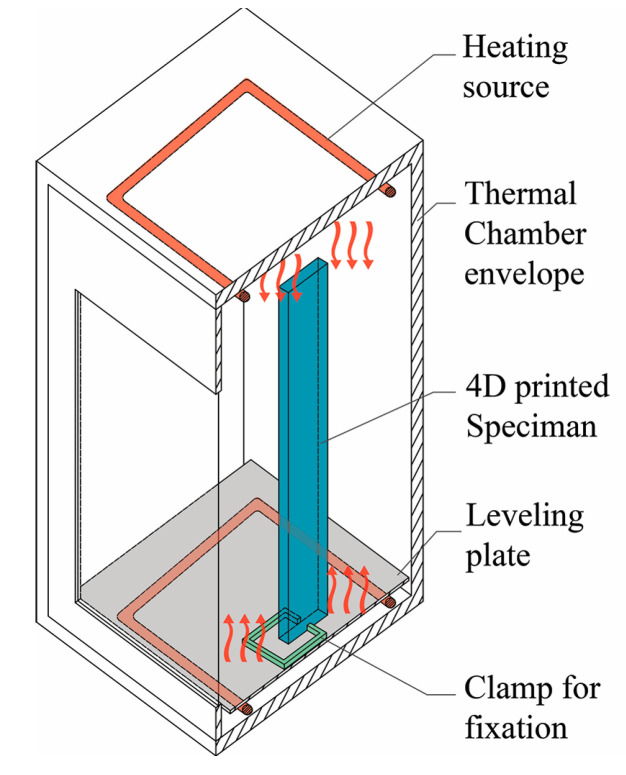
Sketch illustrating uniform heat distribution on 4D-printed sample during thermal programming.

In the thermal stimuli application, 3D-printed samples were heated in an enclosed chamber to ensure uniform heat distribution on samples. During the experiment, each sample was fixed in place using a clamp at the bottom part and left free hanging upward, as shown in Figure 2. A thermometer was placed during the heating process to determine the temperature range at which actuation occurs. At the actuation temperature of samples, the oven door was opened to allow the samples to cool to room temperature and prevent excess heating. Experiments were video recorded. Actuation and angle of curvature were determined using Kinovea software by analyzing the recorded physical experiment images. The images were recorded every 5 s.

The experimental model consisted of six major steps: form design, infill variation design, FDM 3D printing, thermal enclosure test, angle of curvature analysis and deducing motion direction. The investigation of method validity was carried out in three tests. In the first test, the method was applied to different materials to investigate the programmability of each while maintaining constant infill parameters for all printed samples, as found in Section 3.1. The following step was to identify the impact of varying the print path parameters on the most programmable material. The second step was divided into two tests, variation of the infill pattern and variation of infill percentage of the bottom and lower layers, as found in Section 3.2.

In the infill percentage variation test, the investigation parameters selection depended on acquiring different overall percentages of the same 4D printing material, which is further explained in Section “3D Printing”. The overall percentages selected provided a wide spectrum of iterations for testing to deduce resulting behavior.

## 3. Experimental Section

### 3.1. 3D Printing by Variable Printing Infill

#### 3.1.1. Variable Infill Layering

**Figure 3:**
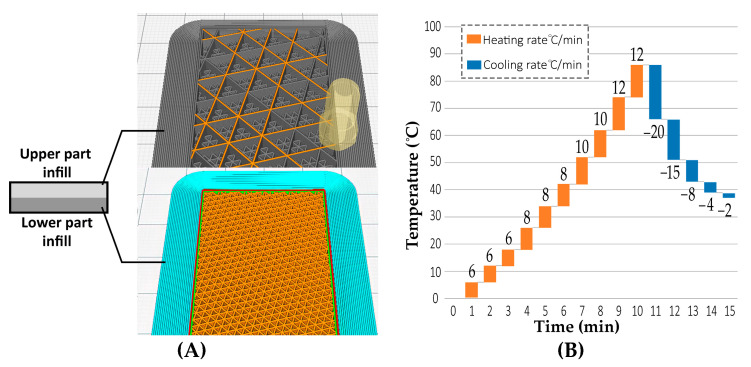
(**A**) Form Iteration 1: hypothesis validity sample modeling. Lower part (Layer 1) of 80% infill and upper (Layer 2) of 20% infill. Simulation of print paths of infill variation using Cura software between layers from bottom to top. (**B**) Graph illustrating heating and cooling rates during thermal programming of 4D-printed TPU.

Samples were designed in the form of a strip with dimensions of 200 × 30 × 6.4 mm^3^, as Form Iteration 1. The strip was equally divided into lower and upper parts: the lower part of 80% infill and the upper part of 20% infill. Each layer had a thickness of 3.2 mm. The variation of print paths, which differed in infill percentage and patterning in the printed layers, is illustrated in Figure 3A. There was an upper layer above the upper part and a lower layer below the lower part. Both the upper and lower layers were fully enclosed, and no gaps between the horizontal layers were found. Additionally, there is no enclosed layer between the upper and lower infills. The overall enclosed perimeter (wall parameter) of the specimen was printed with a thickness of 0.4 mm. PLA+, TPU, PETG and PA6 were 3D printed with the material distribution design of Form Iteration 1. All printed samples were of a diagonal infill pattern. Each sample was tested in the thermal enclosure separately.

The experimental thermal stimuli application test was conducted. The graph in Figure 3B illustrates the relationship between time and the thermal cycle heating and cooling rates. The cooling rate exceeded 16 °C/min for the PLA, PETG and TPU samples. In the PA6 sample, the cooling rate was 2 °C/min.

#### 3.1.2. Results

**Figure 4:**
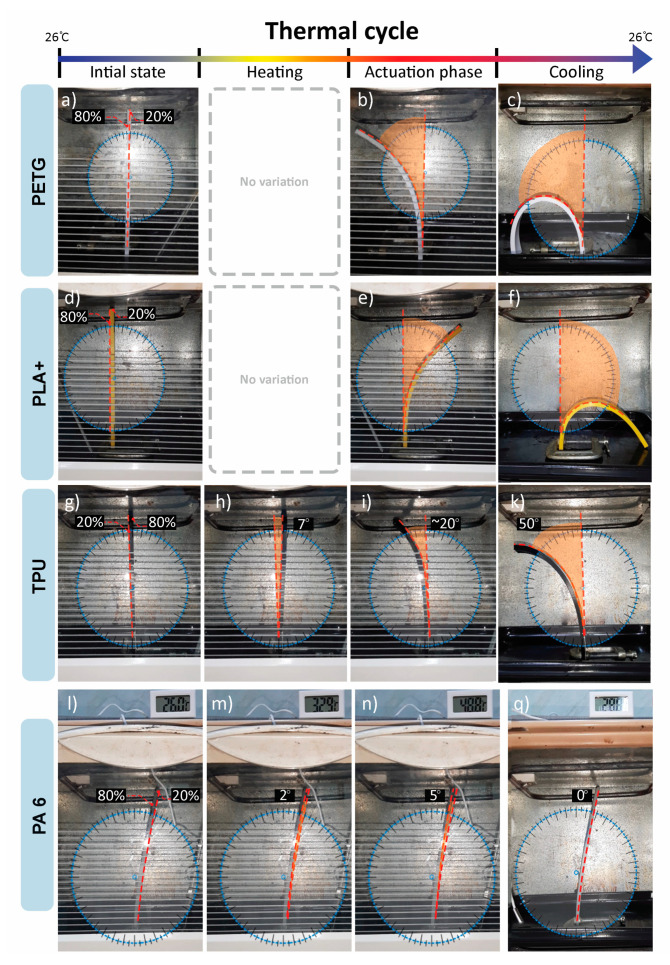
Comparison between PETG, PLA+, TPU and PA6 thermal test results using Kinovea to determine angles of curvature: (**a**) initial form of PETG; (**b**) actuation above 60 °C of 30° curvature, then 60° at 85 °C in 80% infill direction; (**c**) total curvature of 180° after cooling; (**d**) initial form of PLA+; (**e**) actuation above 65 °C of gradient curvature; (**f**) total curvature of 180° after cooling; (**g**) initial form of TPU; (**h**) 7° recoverable curvature toward 80% infill; (**i**) actuation at temperature ~80 °C, deflection toward 20% infill direction; (**k**) 50° total curvature in 80% infill direction after 2° recovery toward 20% infill; (**l**) initial form of PA 6; (**m**) actuation temperature above 28 °C toward 80% infill of 2° curvature; (**n**) actuation temperature above 40 °C toward 80% infill of 3° curvature; (**q**) 5° recovery in 20% infill direction.

PLA+ and TPU show total deflection in the direction of the lower infill percentage. PETG shows total deflection in the higher infill layer direction, while PA 6 shows a recoverable curvature toward the higher infill, as shown in Figure 4. PLA+, TPU and PETG show gradient angles of curvature after actuation temperatures of 65 °C, ~80 °C and ~60 °C, respectively as shown in Figure 4c,e,h–k. PA 6 shows variable curvature angles at a lower actuation temperature of 28 °C, observed in the first step of actuation Figure 4m. PETG and PLA+ show no recovery to initial position during cooling and no recovery after stimuli removal Figure 4c,f. TPU shows a recoverable 7° curvature during the test toward a higher infill percentage layer above 40 °C, which presents the first step of actuation Figure 4h. It then deflects in the lower infill direction of 59°, which is the second step of actuation at a temperature of 108 °C, and after cooling to room temperature, it recovers by 2°. The total attained curvature is 50° Figure 4k. TPU deflection toward a higher infill is observed to be recoverable shape memory upon stimuli removal. PA6 deflects in the 80% infill direction for 2° above 28 °C Figure 4m, then at 3° above 34 °C Figure 4n. PA6 shows total recovery to the original position during cooling Figure 4q.

### 3.2. Programming Variations Using 3D Printing Infill Parameters

#### 3.2.1. Infill Pattern Parameter

**Figure 5:**
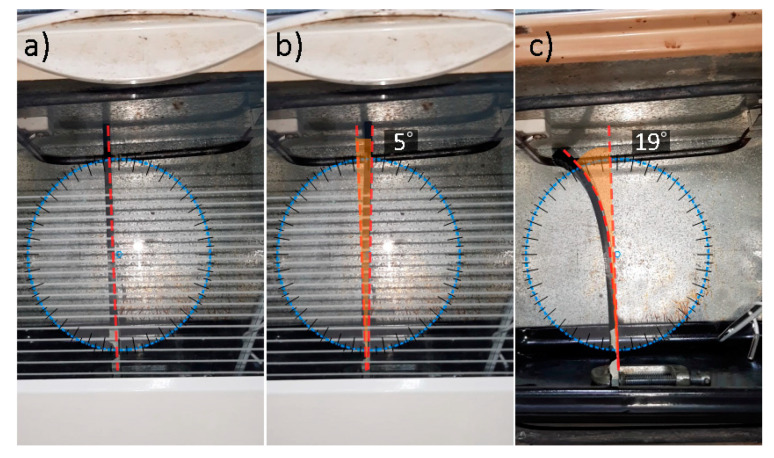
TPU sample of grid infill pattern: (**a**) initial position before heating; (**b**) deflection toward higher infill percentage; (**c**) total curvature after cooling toward lower infill percentage.

The pattern variance effect on shape shifting was tested by comparing the angle of deflection of the TPU sample of the same spatial dimensions and two different pattern iterations, which were grid and diagonal infill patterns. A TPU sample with dimensions of 200 × 30 × 6.4 mm was 3D printed with a grid infill pattern, different from the diagonal 3D-printed pattern of the TPU sample in Section 3.1.2. As shown in Figure 5, upon heating, the grid infill pattern sample deflects in the 80% infill direction for 5° recovery Figure 5b, then deflects back in the 20% infill direction for ~25°, with a net angle of curvature of 19° Figure 4c. The total angle of curvature of the grid infill pattern is ~31° less than the diagonal infill sample.

#### 3.2.2. Infill Percentage Parameter

This section investigates the impact of varying the infill percentage of each layer on the shape-shifting behavior upon stimuli application and the resultant variance of the angle of curvature. Based on the previous results in Figure 4, it was deduced that the 3D-printed TPU shows the largest angle of curvature using the variable infill method. Thus, it was selected to investigate the infill percentage parameter effect. Samples were printed with a more variable infill percentage for each layer than in Figure 3A. Each sample was subjected to a heating cycle of ramping the thermal enclosure to 110 °C, then the enclosure was opened to allow free cooling to reach room temperature. The room temperature was kept at 25 °C.

##### 3D Printing

The printed samples were of a diagonal infill pattern because, from the infill pattern variance test experiment, it was found that the diagonal pattern allows larger deformation. Samples of Form Iteration 1 have overall dimensions and layer thickness. Six different infill percentage iterations were 3D printed to investigate the material behavior. The iterations were designed by varying the percentages of the upper part’s infill percentage with a constant lower infill percentage of 80%. The overall percentage was the ratio between the upper- and lower-part infill percentages in Equation (2), and the overall percentages tested were 10%, 20%, 25%, 30%, 40% and 50%.

(2) $$Overall\;percentage=\frac{Upper\;Part\;infill\;percentage}{Lower\;Part\;infill\;percentage}$$

Verifying the effect of the overall percentages of the variable 3D-printed infills—upper- to lower-part infill percentages—the impact on curvature was measured by testing the same overall percentages with another constant lower infill percentage of 60%, as shown in Table 1. Three samples of each infill percentage iteration were tested separately, and the resulting angles of the curvatures of samples of the same variable infill iteration were of a 0–1° curvature difference.

##### Results

In all samples, deflection toward the higher infill percentage was deduced at the beginning at a temperature above ~40 °C, and following actuation toward a lower infill percentage was detected above ~80 °C. All samples of the 80% lower infill showed maximum curvature at 110 °C, while all samples of 60% lower infill showed maximum curvature at 108 °C.

**Figure 6:**
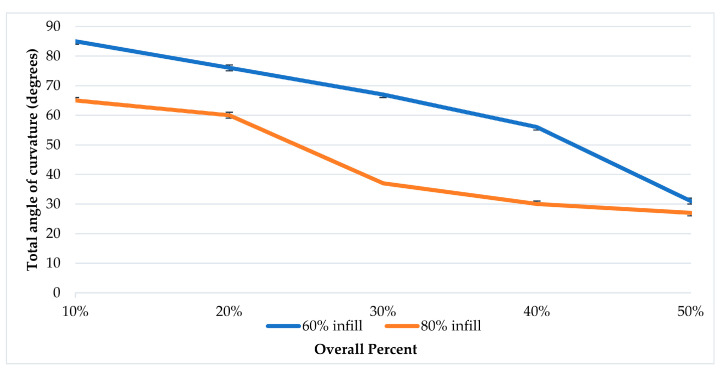
Overall percentage and resulting programming curvature graph. Deviations range from 0 to 1 degrees of curvature.

As shown in Table 1, in samples of the 60% lower-layer infill, the 60–6% infill sample shows 85°, the 60–12% infill sample shows 76°, the 60–15% infill sample shows 72°, the 60–18% infill sample shows 67°, the 60–24% infill sample shows 56° and the 60–30% infill sample shows 31°. In the samples of the lower layer of 80% infill, the 80–8% infill sample shows 65°, the 80–16% infill sample shows 60°, the 80–20% infill sample shows 50°, the 80–24% infill sample shows 37°, the 80–32% infill sample shows 30° and the 80–40% infill sample shows 27°. In Figure 6, a graph is deduced from the empirical experiment results. The graph represents the relationship between the sample overall percentage and the resulting curvature due to thermal stimuli.

### 3.3. Differential Scanning Calorimetry (DSC) Test

**Figure 7:**
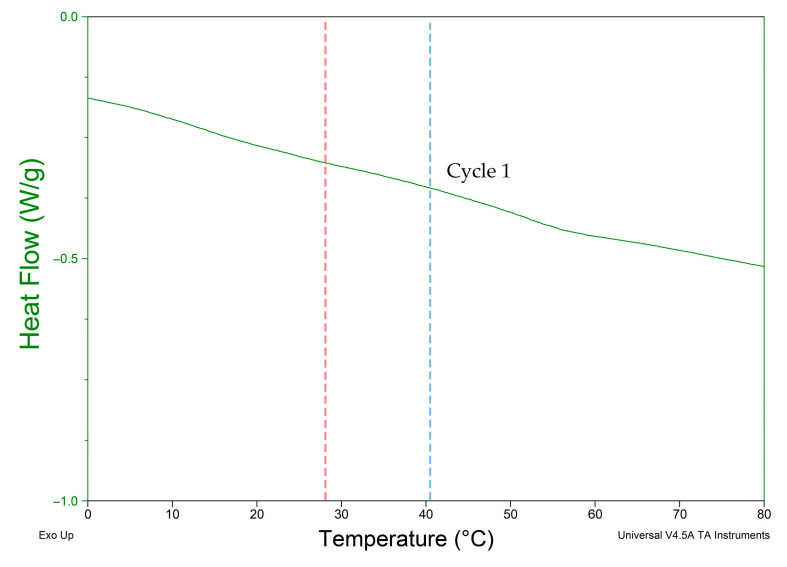
DSC test result of PA 6 3D-printed sample. Red line indicates first actuation step. Blue line indicates second actuation step.

**Figure 8:**
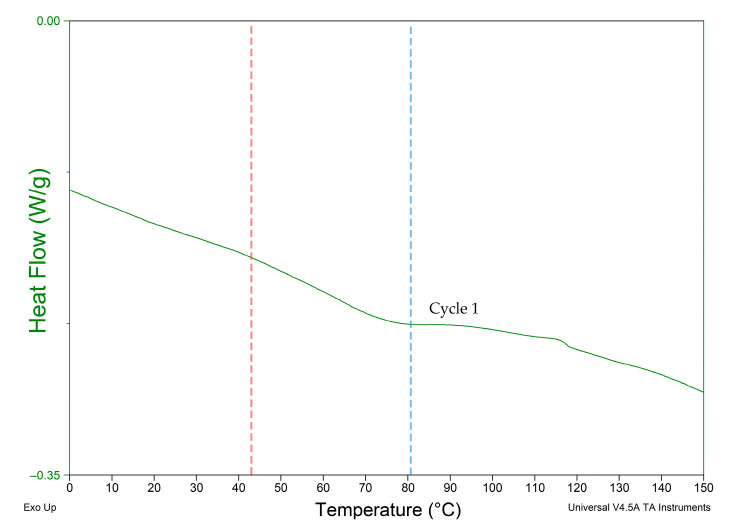
DSC test result of TPU. Red line indicates first actuation step. Blue line indicates second actuation step.

TA Instrument Q2000 DSC (Mettler Toledo, Columbus, OH, USA) was used on materials showing programmability. The test was used to identify the thermal properties of TPU 95A and PA 6 to investigate the observed infill variation experiment results with the thermal properties. A cyclical heat–cool–heat pan test was applied to the polymeric TPU 95A sample with a weight of 10 mg and PA6 with a weight of 6.5 mg. Each sample was heated at a rate of 10 °C/min from −50 °C up to 350 °C under nitrogen atmosphere. Three cycles were registered for each sample, as shown in Figure 7 and Figure 8, in which Cycle 1 represents the thermal properties of the material during the experimental tests.

## 4. Discussion

Shape-shifting behavior was investigated by programming a 4D-printed mono-material with variable parameters of infill percentage and pattern. Infill percentage variation between the top and bottom parts of the sample was observed to control the total angle of curvature and motion direction. Upon investigation of the four polymers, it was deduced that PA 6 and TPU 95A show programmability with the proposed 4D printing method. The broad glass transition indicates the crystalline nature of PA 6 and TPU 95A. PETG and PLA are known to have an amorphous structure [31,32]. Although PA 6 has a lower elongation percentage and a higher Young’s modulus than PETG, PA 6 shows recoverable programmability, while PETG does not, as shown in Figure 4. Figure 7 and Figure 8 show that PA 6 and TPU 95A have broader glass transition ranges than PLA+ and PETG. The results present that in the low range of polymers’ Young’s modulus, the selection criteria of the programmable material of the mono-material 4D printing method can be referred to as the crystallinity of PA 6 and TPU 95A.

**Figure 9:**
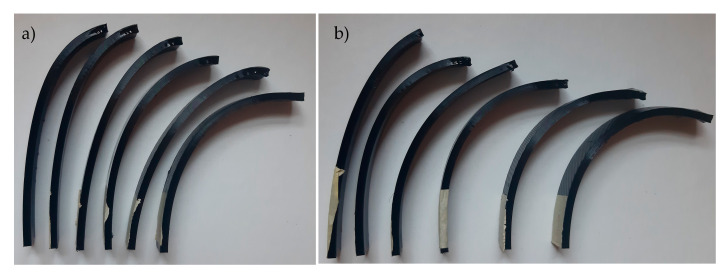
(**a**) Lower layer of 80% infill. Upper-layer infill percentages from left to right: 32%, 24%, 16% and 8%. (**b**) Lower layer of 60% infill. Upper-layer infill percentages from left to right: 32%, 18%, 15%, 12% and 6%.

When analyzing the behavior of programmed materials using the mono-material 4D printing method, one-way and two-way shape-memory effects (SMEs) are observed. PA 6 shows two recoverable actuation steps, and the temperatures of actuation lie within the glass transition range, as shown in Figure 7. The one-way SME of PA 6 is observed upon exceeding the glass transition range. The 4D-printed TPU can be programmed as a one-way and two-way SMP. The 4D-printed TPU shows curvature toward a higher infill percentage, which is totally recoverable upon stimuli removal on the condition that the programming temperature does not exceed 80 °C as the first step of actuation. One-way 4D printing of TPU results upon exceeding 80 °C and the control of the cooling time and rate. One-way 4D-printed TPU results in the second step of actuation. The infill percentage variance in Figure 6 shows that higher angles of curvature result from samples of a constant 60% rather than 80% bottom-part infill. By comparing the behavior recorded of TPU in Figure 9 with the thermal properties graph in Figure 8, the first step of recoverable actuation is within the glass transition range, while the second step of actuation exceeds the glass transition range. Figure 9 indicates that gradient degrees of curvature can result by applying the same stimuli to several TPU 95A samples of a variable overall infill percentage. Angles of curvature deduced with 80% lower part infill in Figure 9a were lower than 60% in Figure 9b. A general behavior is deduced which is the lower the bottom-layer infill percentage, the higher the degree of curvature.

The mono-material 4D printing method presents an upscaling approach. Programming of the 4D-printed components depends on stresses generated within the same 3D-printed layers of the material, which differ from studies in the literature on multi-material 4D printing to achieve stresses in the contact zones between different materials as motion drivers upon stimuli application. In the literature, multi-materials have mostly been actualized by two separate processes of manufacturing, unlike this paper, which proposes a single 4D printing process. It is noted that although Galdman et al.’s method used single filament for 4D printing, it depended on the allocation of fibrils inside the printed filament for directional motion control; thus, directional motion programming was performed during the fabrication step of the filament as a first step, followed by the patterning effect during 3D printing. The 4D printing method presented in this research utilizes single-material 4D printing, where the directional motion programming step is performed during 3D printing only. The plasticizing effect helps control the T_g_**,** which presents a method of investigation to achieve 4D printing components that can actuate within the built environment temperature range. The exposure of the component to the built environment can be investigated to validate the efficiency of the plasticizing effect in 4D printing architecture products. Additionally, integration of fillers in the presented 4D printing method can be investigated to enhance the efficiency of the 4D-printed component performance.

By investigating the application in architecture products, it is found that the interactive architecture system consists of sensors, actuators and a movable feature. Two-way PA 6 observed actuation temperatures lie within the built environment temperature range. Two-way TPU 95A recoverable actuation lies in the higher built environment temperature range, which can offer applications in arid climates. Thus, exploiting recoverable 4D-printed PA 6 and TPU 95A as alternatives to sensors and actuators while using renewable energy—solar radiation—as the motion driver would reduce electric energy consumption for shape-shifting façade systems, as well as reduce system pieces production. Adding the aspect of reduced production to sustainable resource consumption—electric energy consumption—would comply with the sustainable development goals of sustainable consumption and product SDGs. Another possible application would be applying one-way 4D-printed components to special tensegrity deployable architecture structures.

## 5. Conclusions

This paper validates the applicability of mono-material 4D printing to acquire directional motion control. The experimental tests indicate the possibility of one-way and two-way 4D printing using mono-material FDM. It is observed that the infill variation method produces a two-way SMP when applying thermal stimuli within the glass transition range, while a one-way SMP is produced by exceeding that range. It is concluded that polymeric filaments of broader glass transition range such as PA 6 and TPU 95A allow programming using the mono-material 4D printing method. The infill parameters of percentage and pattern affect the acquired angle of curvatures upon thermal stimuli application. A diagonal infill pattern allows a larger resulting angle of curvature upon stimuli application. It is deduced from Table 1 that the lower the overall upper- to lower-part infill percentages, the higher the angle of curvature. Additionally, it is concluded that the lower the infill percentage of the top part, the higher the resulting angle of curvature.

## Figures and Tables

**Table 1:** Infill percentage parameter test results. All samples show net curvature deflection toward the lower infill percentage part.

Diagonal Infill Pattern					
Overall Percent	Lower-Layer Infill %	Upper-Layer 2 Infill %	Total Angle of Curvature	Net Motion Direction Toward	Recoverable Deflection Angle toward Higher Infill %
10%	80	8	65°	Lower infill	7°
20%	80	16	60°		7°
25%	80	20	50°		7°
30%	80	24	37°		5°
40%	80	32	30°		7°
50%	80	40	27°		7°
10%	60	6	85°	Lower infill	5°
20%	60	12	76°		7°
25%	60	15	72°		5°
30%	60	18	67°		5°
40%	60	24	56°		5°
50%	60	30	31°		5°
